# NLRC5 Deficiency Deregulates Hepatic Inflammatory Response but Does Not Aggravate Carbon Tetrachloride-Induced Liver Fibrosis

**DOI:** 10.3389/fimmu.2021.749646

**Published:** 2021-10-12

**Authors:** Akouavi Julite I. Quenum, Akhil Shukla, Fjolla Rexhepi, Maryse Cloutier, Amit Ghosh, Thomas A. Kufer, Sheela Ramanathan, Subburaj Ilangumaran

**Affiliations:** ^1^ Department of Immunology and Cell Biology, Faculty of Medicine and Health Sciences, Université de Sherbrooke, Sherbrooke, Canada; ^2^ Department of Immunology (180b), Institute of Nutritional Medicine, University of Hohenheim, Stuttgart, Germany; ^3^ Centre de Recherche du Centre Hospitalier Universitaire de Sherbrooke (CR-CHUS), Sherbrooke, Canada

**Keywords:** NLRC5, NF-κB, liver fibrosis, carbon tetrachloride, hepatic stellate cells

## Abstract

The nucleotide-binding leucine-rich repeat-containing receptor (NLR) family protein-5 (NLRC5) controls NF-κB activation and production of inflammatory cytokines in certain cell types. NLRC5 is considered a potential regulator of hepatic fibrogenic response due to its ability to inhibit hepatic stellate activation *in vitro*. To test whether NLRC5 is critical to control liver fibrosis, we treated wildtype and NLRC5-deficient mice with carbon tetrachloride (CCl_4_) and assessed pathological changes in the liver. Serum alanine transaminase levels and histopathology examination of liver sections revealed that NLRC5 deficiency did not exacerbate CCl_4_-induced liver damage or inflammatory cell infiltration. Sirius red staining of collagen fibers and hydroxyproline content showed comparable levels of liver fibrosis in CCl_4_-treated NLRC5-deficient and control mice. Myofibroblast differentiation and induction of collagen genes were similarly increased in both groups. Strikingly, the fibrotic livers of NLRC5-deficient mice showed reduced expression of matrix metalloproteinase-3 (*Mmp3*) and tissue inhibitor of MMPs-1 (*Timp1*) but not *Mmp2* or *Timp2*. Fibrotic livers of NLRC5-deficient mice had increased expression of TNF but similar induction of TGFβ compared to wildtype mice. CCl_4_-treated control and NLRC5-deficient mice displayed similar upregulation of *Cx3cr1*, a monocyte chemoattractant receptor gene, and the *Cd68* macrophage marker. However, the fibrotic livers of NLRC5-deficient mice showed increased expression of F4/80 (*Adgre1*), a marker of tissue-resident macrophages. NLRC5-deficient livers showed increased phosphorylation of the NF-κB subunit p65 that remained elevated following fibrosis induction. Taken together, NLRC5 deficiency deregulates hepatic inflammatory response following chemical injury but does not significantly aggravate the fibrogenic response, showing that NLRC5 is not a critical regulator of liver fibrosis pathogenesis.

## Introduction

Fibrotic diseases of the liver, as well as that of other organs such as lungs, kidneys, heart and pancreas, arise from chronic inflammation that causes perpetual tissue damage (1). Persistent inflammation deregulates the tissue repair process and leads to progressive replacement of the parenchymatous cells with abnormal extracellular matrix (ECM), which compromises organ functions and necessitates organ transplantation in advanced stages of disease (2). Impressive progress has been made in understanding the cellular components, their secretory products and molecular pathways of fibrogenesis with the goal of finding ways to halt disease progression as well as promote fibrosis resolution and restoration of tissue homeostasis (3–5). Despite the limited success of available treatments targeting various molecules of the fibrogenic signaling pathways, this approach remains the mainstay for finding new strategies to treat fibrotic diseases (6, 7).

Liver fibrosis often results from chronic hepatitis virus infections, alcohol abuse and from obesity-associated fatty liver disease (8–10). Chronic inflammatory stimuli that accompany these conditions induce pro-inflammatory cytokines and chemokines from injured hepatocytes and liver-resident macrophages (Kupffer cells) that promote recruitment of circulating monocytes and their differentiation towards pro-inflammatory macrophages (11, 12). This inflammatory response activates hepatic stellate cells (HSC), which are also directly activated by injured hepatocytes, resulting in HSC proliferation and differentiation towards myofibroblasts that express α-smooth muscle actin (αSMA) (13). Growth factors and the profibrogenic cytokine transforming growth factor beta (TGFβ) secreted by pro-inflammatory macrophages induce fibroblast proliferation and ECM deposition to facilitate wound healing and tissue repair. Pro-resolution macrophages also produce ECM remodeling enzymes such as matrix metalloproteinases (MMP) to resolve the fibrous scar tissue. However, incessant inflammatory stimuli establish a feed forward loop of pro-inflammatory and pro-fibrogenic processes (4). Progressive replacement of the liver parenchyma with fibrous scar tissue results in an end-stage disease called cirrhosis (9, 11). In addition to being a major cause of global healthcare burden and mortality, cirrhosis promotes the development of hepatocellular carcinoma (HCC), one of the most common and lethal cancers worldwide (14–18). HCC takes decades to present clinical symptoms and is often diagnosed in late stages, for which there are very few therapeutic options (19). As most HCC cases arise from cirrhotic livers, therapeutic targeting of molecules and cells that promote hepatic fibrogenesis is considered a promising avenue to halt HCC development and progression, in addition to improving liver functions (20–23).

Members of the nucleotide binding and oligomerization domain (NOD)-like receptors (NLRs) constitute a family of cytosolic pattern recognition receptors that play a key role in inflammatory responses (24). The NLR proteins are further classified based on their N-terminal domains into NLRA, NLRB, NLRC and NLRP subgroups, each with one or more members, and most of them harboring C-terminal leucine-rich repeats (24, 25). Whereas certain members of NLRP (NLRP1, NLRP3) and NLRC (NLRC4) subfamilies activate inflammasomes and induce production of pro-inflammatory cytokines IL-1β and IL-18, certain members of the NLRC family (NOD-1, NOD-2) activate the nuclear factor kappa-light-chain-enhancer of activated B cells (NF-κB) to induce the expression of genes coding for these pro-inflammatory cytokines (24, 26). NLRA and NLRC5 function as transcriptional activators of MHC class-II and class-I genes, respectively, and thus are respectively known as class-II transactivator (CIITA) and class-I transactivator (CITA) (27). NLRC5 has also been implicated in regulating inflammatory response similarly to NLRC3 and NLRX1, both of which contain poorly defined N-terminal domains (24, 28–33). Over expression and knockdown studies have shown that NLRC5 inhibited LPS-induced NF-κB activation and induction of TNFα, IL-6, RANTES (CXCL5) genes and IL-1β secretion (28, 29, 34).

Given the prominent role of inflammatory cytokine signaling in liver fibrosis and TNFα-induced NLRC5 expression in the human HSC cell line LX-2, Li and colleagues investigated the role of NLRC5 in modulating the fibrogenic response in HSCs (35–37). Stable NLRC5 expression in LX-2 cells was shown to increase TNFα-induced IL-6 and IL-1β mRNA expression, whereas siRNA-mediated NLRC5 knockdown diminished this response, although these effects did not affect IL-6 or IL-1β protein expression (35). This study also reported that NLRC5 knockdown increased TNFα-induced IκB phosphorylation, nuclear localisation of the p65 component of NF-κB and phosphorylation of SMAD3, a key transcription factor activated by the profibrogenic cytokine TGFβ, suggesting an anti-fibrogenic role for NLRC5 (35). The same group also reported elevated NLRC5 expression in human fibrotic livers and that stable NLRC5 expression in LX-2 cells upregulated TGFβ-mediated induction of αSMA and collagen 1α1 (36). However, knockdown of NLRC5 was shown to increase TGFβ-mediated apoptosis of LX-2 cells despite increasing the phosphorylation of NF-κB, SMAD2 and SMAD3 (36). Following experimental hepatic fibrogenesis in C57BL/6 mice, increased NLRC5 expression was observed in the fibrotic livers that coincided with collagen 1α1 and αSMA expression and all three genes showed diminished expression during fibrosis resolution (37). Inhibition of LX-2 cell activation by a mixture of methylxanthine, dexamethasone and insulin, which inhibits TGFβ-mediated upregulation of αSMA and collagen 1α1 also inhibited NLRC5 induction in LX-2 cells (37). Based on these findings, Li and colleagues proposed an anti-fibrogenic role for NLRC5 in a negative feedback manner, following its induction in HSCs by TNFα and TGFβ. Here, we sought genetic evidence for this hypothesis by evaluating liver fibrosis induced by carbon tetrachloride (CCl_4_) in NLRC5-deficient mice.

## Methods

### Mice

*Nlrc5^-/-^* mice in C57BL/6N background, generated by crossing *Nlrc5-floxed* mice with CMV-Cre mice, were a generous gift from Dr. Dana Philpott (38). Wildtype C57Bl/6N mice were used as controls. Both groups of mice were bred and housed in ventilated cages on the same housing unit throughout the experiment. The experiments were done as and when the knockout mice became available. Therefore, the numbers of mice used per group in different experiments was variable and are indicated in the corresponding figure legends. All experimental protocols on animals were carried out with the approval of the Université de Sherbrooke Animal Ethics Committee (Protocol # 2018-2083, 359-18C).

### Liver Fibrosis Induction by Carbon Tetrachloride

Liver fibrosis was induced as we have described previously (39). Male mice were used for liver fibrosis induction as female sex hormones diminish inflammatory cytokine production in the liver (40). Briefly, CCl_4_ (Sigma-Aldrich, Oakville, ON) diluted in corn oil (1:3) was injected *via* intraperitoneal (i.p) route (0.5μl CCl_4_ per gram body weight) twice a week for five weeks. Three days after the last treatment, mice were euthanized, blood collected by cardiac puncture and liver tissues resected. Serum was separated and kept frozen at -80°C. Liver pieces were snap frozen and stored at -80°C for gene and protein expression studies and hydroxyproline assay. For histopathology analyses, 3-4 cubic mm size liver pieces from 4-5 different locations of the same liver were fixed for 12-16 hours in 4% paraformaldehyde solution and embedded in paraffin on the same tissue block.

### Serum ALT and Liver Hydroxyproline Assays

Serum alanine transaminase (ALT) levels were measured using a kinetic assay (Pointe Scientific Inc, Brussels, Belgium) following manufacturer’s instructions. Hydroxyproline content was measured as described previously (39). Ten mg of liver tissue, homogenized in 1 mL of 6N HCl using the bead mill MM 400 (Retsch, Hann, Germany), was transferred to glass tubes, topped up with 2 mL of 6N HCl and the tubes were kept on a heat block for 16h at 110°C to hydrolyze proteins. After filtering the hydrolysate through Whatman #1 filter paper, aliquots were evaporated on a heat block and the residues were dissolved in 50% 2-propanol. Hydroxyproline standards and samples, distributed in a 96-well microtiter plate, were oxidized by adding chloramine T (Sigma-Aldrich; dissolved in 50% isopropanol and adjusted to pH 6.5 with acetate/citrate buffer). Following incubation at room temperature for 25 min, Ehrlich reagent [*p*-dimethylaminobenzaldehyde dissolved in n-propanol/perchloric acid (2:1)], was added and the samples incubated at 50°C for 10 min for color development. Absorbance at 550 nm was measured using the SPECTROstar Nano (BMG Labtech, Germany) spectrophotometer.

### Histology and Immunohistochemistry

Liver sections were deparaffinized, rehydrated, and stained with hematoxylin and eosin (H&E) or Sirius red following standard procedures. For immunohistochemical detection of αSMA, rehydrated liver sections immersed in citrate buffer (pH 6.0) were given intermittent microwave treatment to retrieve antigenic epitopes. Following incubation in 3% hydrogen peroxide for 10 min to inhibit endogenous peroxidase activity, sections were blocked with 5% BSA in Tris-buffered saline (TBS) containing 20% Tween-20 (TBS-T). Slides were incubated overnight at 4°C with a rabbit mAb against mouse αSMA (Cell Signaling Technology, Cat #19245S) diluted in blocking buffer, washed and then incubated with horseradish peroxidase (HRP)-conjugated secondary Ab for 1 h. After thorough washing, a substrate solution containing 3,3’-diaminobenzidine (DAB; Sigma-Aldrich; 30 μL chromogen diluted in 1 mL of DAB liquid buffer) was added for 10 min. The sections were counterstained with hematoxylin and mounted with a coverslip. Images of the stained sections, digitized using the NanoZoomer Slide Scanner (Hamamatsu Photonics, Japan), were analyzed by the NanoZoomer Digital Pathology software NDPview2.0. Sirius red staining and αSMA-positive areas were quantified using the NIH ImageJ software (version 1.53e). Data from six randomly selected fields from different liver pieces for each of the three mice per group were used for quantification.

### Gene Expression Analysis

Total RNA from frozen tissues was extracted using QIAzol Lysis Reagent (Qiagen, Toronto, Ontario, Canada), according to the manufacturer’s instructions. cDNA was synthetized from 1µg of purified RNA using QuantiTect^®^ reverse transcription kit (Qiagen, Toronto, Ontario, Canada). Quantitative RT-PCR amplification reactions were carried out in CFX Connect Real-Time PCR Detection System (Bio-Rad, Canada) or QuantStudio 3 Real-Time PCR System (Thermo Fisher Scientific, Canada) using SYBR Green Supermix (Bio-Rad, Mississauga, Ontario, Canada). The expression of indicated genes was measured using primers listed in **Supplementary Table S1**. Gene expression levels between samples were normalized based on the Cycle threshold (Ct) values compared to housekeeping gene *36B4* and the fold induction was calculated using the vehicle (oil)-treated wildtype mice as controls.

### Enzyme Linked Immunosorbent Assay (ELISA)

Serum TNF protein levels were quantified using a sandwich ELISA kit from eBioscience (Cat # 88-7324) following manufacturer’s instructions. Capture Ab diluted in coating buffer was added to high protein-binding 96-well plates (Nunc Maxisorp^®^) and incubated overnight at 4°C. After washing with PBS-0.05% Tween-20 (wash buffer), the plates were blocked with assay diluent for 1 h at room temperature. Serum samples diluted 1:1 in assay diluent and serial dilutions of recombinant TNF standard were added in duplicates, and plates were incubated at room temperature for 2 h. After thorough washing, biotinylated detection antibody was added for 1 h followed by the addition of avidin-HRP for 30 min. After thorough washing, tetramethylbenzidine substrate solution was added for 15 min and color development was measured at 450 nm using SPECTROstar Nano. The values were plotted against the standard curve to calculate TNF protein levels in serum.

### Western Blot

Mice liver tissue samples were taken in a 2 mL round bottom tube and homogenized using bead mill MM 400 (Retsch, Hann, Germany) containing TNE buffer (50mM Tris-HCl, 150mM NaCl, 1mM EDTA; pH 8.0) supplemented with phosphatase and protease inhibitor cocktails (Roche, Indianapolis, IN). TNE buffer containing detergents (0.2% SDS, 1% sodium deoxycholate and 1% Triton-X) was added in equal volumes into the homogenates and kept on rocker for 30 min at 4°C. Lysate was centrifuged for 20 min at 15,000 ×*g* and the supernatant collected. Protein concentration was determined using RC-DC Protein Assay Kit (Bio-Rad, Mississauga, ON). Protein samples containing 30-50 µg proteins were electrophoresed on SDS-PAGE gels and analysed by Western Blot. Primary Ab used are listed in **Supplementary Table S2**. HRP-conjugated anti-mouse or anti-rabbit secondary antibodies and enhanced chemiluminescence reagents (ECL) were from GE Healthcare Life Sciences (Pittsburg, PA). Images of western blot were captured by the VersaDOC 5000 imaging system (Bio-Rad).

### Statistical Analysis

The numbers of mice in experimental and control groups for the two genotypes of mice in each experiment are indicated in corresponding figure legends. Data were analyzed using the GraphPad Prism9 (San Diego, CA). Statistical significance was calculated by two-way ANOVA with Tukey’s post-hoc test. *p* values <0.05 were considered significant.

## Results

### Loss of NLRC5 Does Not Exacerbate Liver Damage Caused by Chemical Injury

**Figure 1:**
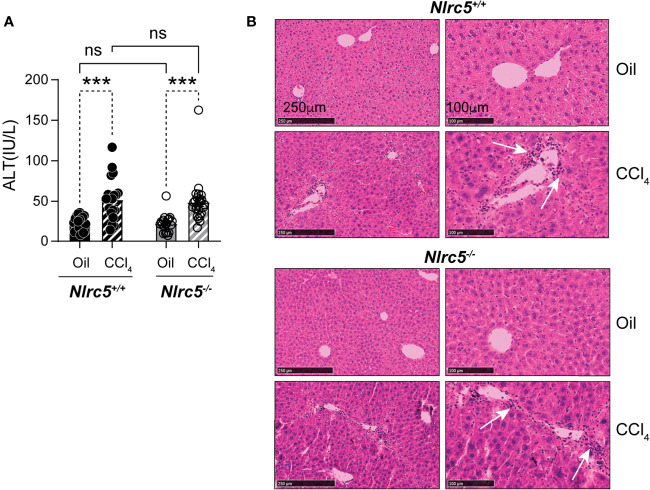
Loss of NLRC5 does not exacerbate liver damage caused by chemical injury. **(A)** Serum ALT levels in NLRC5-deficient and control mice following 5 weeks of treatment with CCl_4_ or corn oil (vehicle). Data shown are mean ± standard error of mean (SEM) from 4-5 mice per group from two separate experiments. Statistical significance was calculated by two-way ANOVA with Tukey’s *post-hoc* test: ***p < 0.001, ns, not significant. **(B)** Images of hematoxylin and eosin-stained sections of the livers, representative of 4-6 mice per group are shown. Magnified images (right) show comparable changes in hepatocyte morphology and mononuclear cell infiltration (arrows) in CCl_4_-treated NLRC5-deficient and control livers.

TNFα, expressed by macrophages and hepatocytes in response to toll-like receptor signaling, contributes to liver fibrosis by activating HSC and immune cells (12). Loss of TNF receptor TNFR1 attenuates liver fibrosis induced by CCl_4_ or bile duct ligation, accompanied by reduced expression of *Col1a1* and *Il6* genes and decreased NF-κB activation in liver tissues as well as in isolated HSCs (41, 42). NF-κB signaling promotes cell survival and proliferation of not only hepatocytes but also HSCs (42–44). As NLRC5 knockdown in HSCs was shown to increase NF-κB signaling (35), we examined whether NLRC5 deficiency promoted liver fibrosis *in vivo*. To this end, we induced liver fibrosis by intraperitoneal administration of CCl_4_ in NLRC5-deficient and control mice for five weeks. Alterations in liver function were evaluated and histological and molecular changes were assessed. As shown in **Figure 1A**, both wildtype and *Nlrc5^-/-^* mice showed comparable levels of liver damage as revealed by elevated serum levels of alanine transaminase (ALT). Hematoxylin and eosin-stained liver sections showed similar features of hepatocyte damage and mononuclear cell infiltration in both wildtype and *Nlrc5^-/-^* mice (**Figure 1B**). Together these results indicated that loss of NLRC5 does not increase hepatocyte damage induced by chronic chemical injury.

### CCl_4_-Induced Liver Fibrosis in NLRC5-Deficient Mice Is Comparable to Wildtype Mice

**Figure 2:**
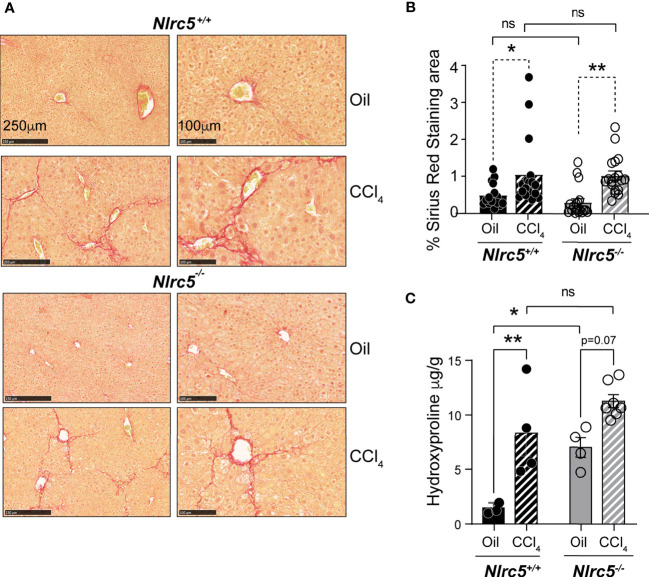
CCl_4_-induced liver fibrosis in NLRC5-deficient mice is comparable to wildtype mice. **(A)** Sirius red-stained sections of oil- or CCl_4_- treated control and NLRC5-deficient livers at low (left) and high (right) magnifications. Data shown are representative of 4-5 mice per group from two independent experiments. **(B)** Quantification of Sirius red-stained area. Six randomly selected fields from liver pieces collected from different locations of each of the three mice per group were used for quantification. **(C)** Hydroxyproline content of livers from oil (n=3-4) or CCl_4_-treated (n=4-7) control and NLRC5-deficient mice. Data shown in **(B, C)** are mean ± SEM. Two-way ANOVA with Tukey’s *post-hoc* test: *p < 0.05, **p < 0.01, ns, not significant.

Next, we compared the extent of liver fibrosis in CCl_4_-treated *Nlrc5^-/-^* and control mice. Sirius red staining of collagen fibers revealed comparable pattern and distribution of fibrotic areas in *Nlrc5^-/-^* and wildtype mice that was also confirmed by quantification of the stained areas (**Figures 2A, B**). Moreover, measurement of hydroxyproline, which is enriched in connective tissue collagen fibers (45), was increased in CCl_4_-treated wildtype mice (**Figure 2C**). Interestingly, *Nlrc5^-/-^* mice treated with vehicle (corn oil, control) showed significantly elevated hydroxyproline content compared to wildtype mice. Because of such elevated hydroxyproline content in *Nlrc5^-/-^* mice, the CCl_4_-mediated increase in this group was not statistically significant, even though these levels are appreciably higher than in CCl_4_-treated wildtype mice (**Figure 2C**). These observations suggested that NLRC5 deficiency may augment certain aspects of the hepatic fibrogenic response that is not discernible in the presence of strong fibrogenic inducers such as CCl_4_.

### CCl_4_-Induced Hepatic Myofibroblast Differentiation Is Similar in NLRC5-Deficient and Wildtype Mice

**Figure 3:**
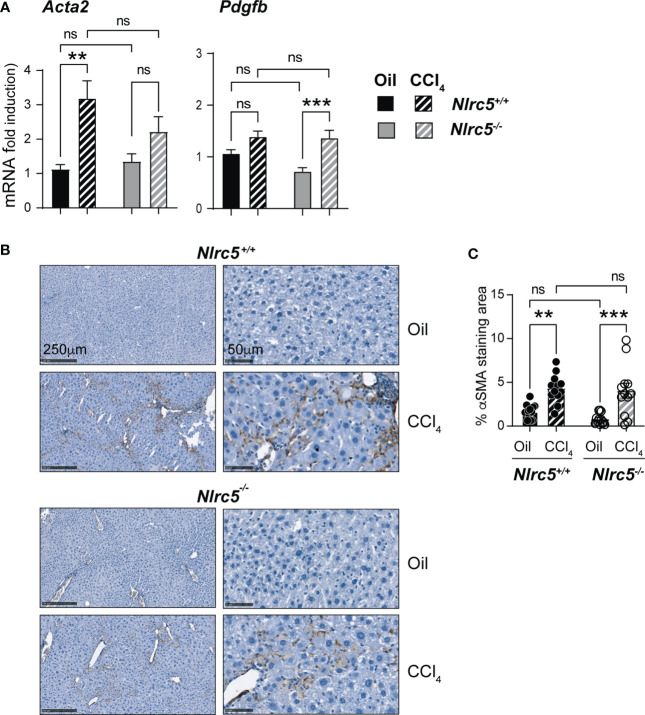
CCl_4_-induced myofibroblast differentiation is similar in NLRC5-deficient and wildtype livers. **(A)** Induction of *Acta2* and *Pdgfb* genes in fibrotic livers. Quantitative RT-PCR analysis of 8-10 mice from two independent experiments. **(B)** Immunohistochemical staining of αSMA in oil- or CCl_4_- treated control and NLRC5-deficient mice livers. Representative liver sections from 4-5 mice per group from two independent experiments are shown. **(C)** Quantification of αSMA-stained areas. Six randomly selected fields from liver pieces collected from different locations of three mice per group were used for quantification. Data shown in **(A, C)** are mean ± SEM. Two-way ANOVA with Tukey’s *post-hoc test:* **p < 0.01; ***p < 0.001; ns, not significant.

As fibrogenesis is mainly driven by HSCs activation and their differentiation to myofibroblasts (13), we evaluated the expression of the *Acta2* gene coding for αSMA and that of *Pdgfb* coding for platelet-derived growth factor beta, a mitogen for HSC. The induction of *Acta2* was significantly high in CCl_4_-treated wildtype mice livers but showed only marginal increase in *Nlrc5^-/-^* mice. On the other hand, *Pdgfb* upregulation was significantly elevated in the livers of CCl_4_-treated *Nlrc5^-/-^* mice but less prominently in control mice (**Figure 3A**). However, the upregulation of *Acta2* and *Pdgfb* genes was not significantly different between CCl_4_-treated wildtype and *Nlrc5^-/-^* mice. Moreover, immunohistochemical staining of αSMA in the liver sections from vehicle- or CCl_4_- treated mice showed a comparable increase in pattern and staining of myofibroblast distribution in CCl_4_- treated wildtype and *Nlrc5^-/-^* mice that was also confirmed by digital quantification of the stained areas (**Figures 3B, C**). These findings indicated that NLRC5 deficiency does not markedly affect myofibroblast differentiation during chemically induced liver fibrosis.

### Similar Induction of Collagens but Differential Induction of ECM Remodelling Enzymes in NLRC5-Deficient and Control Livers

**Figure 4:**
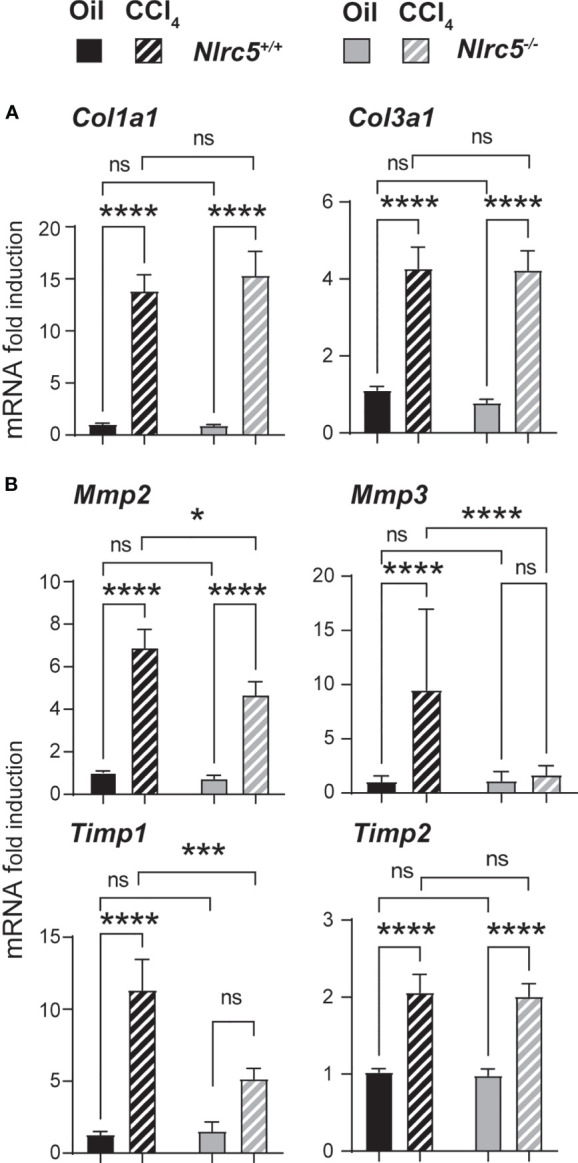
Similar induction of collagens but differential induction ECM remodelling enzymes in NLRC5-deficient and control livers. RNA extracted from liver tissues from the indicated groups of mice were evaluated for the expression of **(A)** collagen (*Col1a1, Col3a1*) and **(B)** ECM remodelling enzymes (*Mmp2, Mmp3, Timp1, Timp2*) by qRT-PCR. Data shown are mean ± SEM; n= 6-10 mice for each group collected from 2-3 independent experiments. Two-way ANOVA with Tukey’s *post-hoc* test: *p < 0.05; ***p < 0.001; ****p < 0.0001; ns, not significant.

Consistent with the comparable levels of myofibroblast differentiation in *Nlrc5^-/-^* and wildtype mice livers following CCL_4_ treatment, genes encoding the fibrillar collagens, collagen 1α1 and collagen 3α1 (46) were strongly induced in both groups (**Figure 4A**). Similarly, the gene coding for the ECM modifying enzyme MMP2 and tissue inhibitor of MMPs-2 (*Mmp2*, *Timp2*), which respectively exert anti- and pro-fibrogenic roles in liver fibrosis (47–49), were strongly upregulated by CCL_4_ treatment in both *Nlrc5^-/-^* and control mice livers (**Figure 4B**). However, *Mmp3* and *Timp1* genes, whose impact on liver fibrosis is controversial or unclear (49), were strongly induced in wildtype mice livers but showed significantly lower or negligible induction in NLRC5-deficient livers (**Figure 4B**). These findings indicate that NLRC5 deficiency does not appreciably affect the induction of many fibrogenic response genes and that the observed differences caused by NLRC5 deficiency are not strong enough to influence the severity of liver fibrosis.

### Fibrotic Livers of NLRC5-Deficient Mice Show Increased TNF Expression

**Figure 5:**
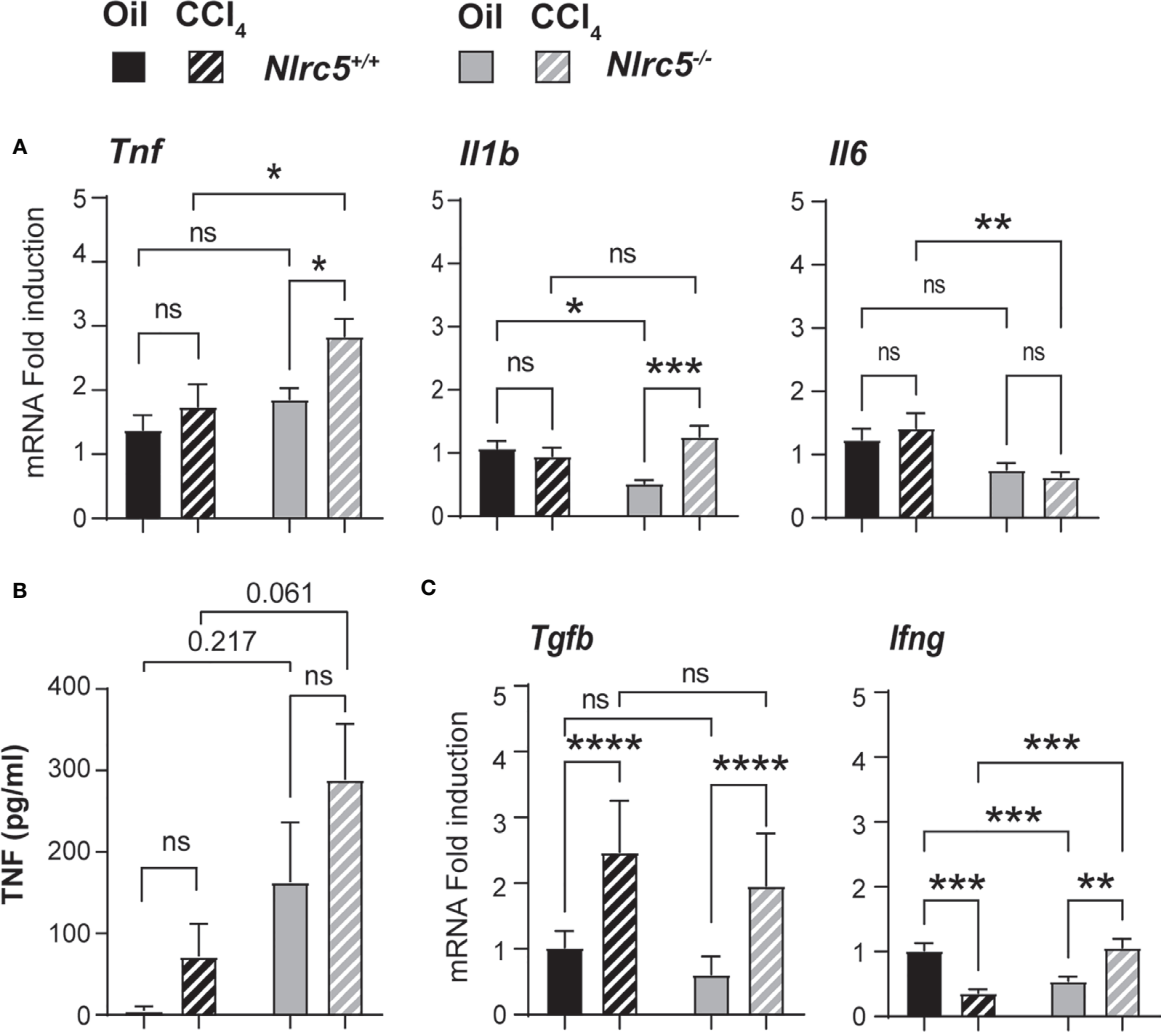
Fibrotic livers of NLRC5-deficient mice show increased TNF expression. **(A)** Hepatic RNA from the indicated groups of mice were tested for the expression of pro-inflammatory cytokine genes *Tnf, Il1b* and *Il6* by qRT-PCR; n= 7-11 mice for each group from 2-3 independent experiments. **(B)** ELISA quantification of serum TNF levels; n=4 mice per group. **(C)** Expression of pro-fibrogenic (*Tgfb*) and anti-fibrogenic (*Ifng*) cytokine genes in the liver tissue samples used in **(A)**. Data shown are mean ± SEM; Two-way ANOVA with Tukey’s *post-hoc* test: *p < 0.05; **p < 0.01; ***p < 0.001; ****p < 0.0001; ns, not significant. For certain comparisons, significance values are indicated.

Liver fibrosis establishes feed forward loops involving pro-inflammatory and profibrogenic cytokine gene expression by immune cells and their recruitment by chemokines (50, 51). To determine how NLRC5 deficiency affects these processes, we first evaluated the expression of candidate genes implicated in these processes. NLRC5-deficient livers displayed a significantly higher induction of the pro-fibrogenic tumor necrosis factor gene *Tnf* (**Figure 5A**). Serum TNF levels were elevated in both control and *Nlrc5^-/-^* mice following CCl_4_ treatment (**Figure 5B**). Notably, vehicle-treated *Nlrc5^-/-^* mice displayed appreciably higher levels of TNF than control mice. The interleukin-1β gene *Il1b* did not show appreciable induction following CCl_4_ treatment in control livers but was significantly elevated in NLRC5-deficient livers due to lower expression in the oil-treated group (**Figure 5A**). The transcript levels of IL-6, a survival cytokine, was appreciably lower in *Nlrc5^-/-^* livers (**Figure 5A**). The *Tgfb* gene coding for the key fibrogenic cytokine transforming growth factor beta showed comparable upregulation in both groups following CCl_4_ treatment (**Figure 5C**). On the other hand, the antifibrogenic interferon gamma gene *Ifng* was appreciably reduced in wildtype livers following CCl_4_ treatment, whereas *Nlrc5^-/-^* livers showed a significant upregulation (**Figure 5C**). These findings indicate that NLRC5 deficiency did cause an upregulation of hepatic *Tnf* gene expression and systemic TNF protein levels, but this did not result in increased liver fibrosis.

### Increase inF4/80 Positive Cells in NLRC5-Deficient Livers

**Figure 6:**
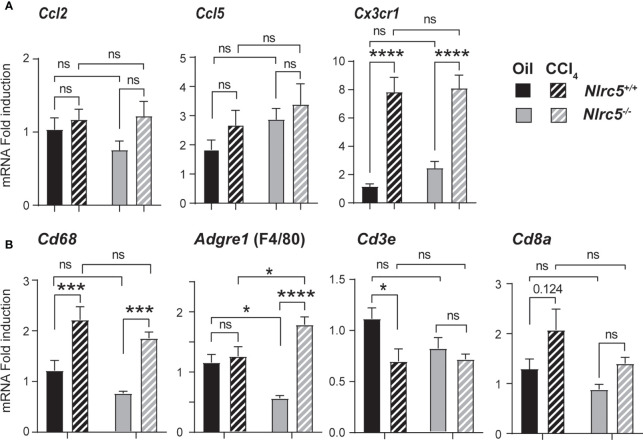
Increased expression of F4/80 gene in the fibrotic livers of NLRC5-deficient mice. RNA extracted from liver tissues from the indicated groups of mice were evaluated for the gene expression of **(A)** chemokines (*Ccl2, Ccl5, Cx3cr1*) and **(B)** the markers of macrophages (CD68, F4/80) and T lymphocytes (CD3ϵ, CD8α). Data shown are mean ± SEM; n= 7-11 mice for each group from 2-3 independent experiments. Two-way ANOVA with Tukey’s *post-hoc* test: *p < 0.05; ***p < 0.001; ****p < 0.0001; ns, not significant. For certain comparisons, significance values are indicated.

The key producer cells of TNF during liver fibrosis are activated liver-resident Kupffer cells and monocyte-derived macrophages, which are recruited by chemokines expressed in the inflamed liver (51). As NLRC5-deficient mice showed elevated TNF expression, we evaluated the gene expression of the macrophage recruiting chemokine CCL2 (macrophage chemoattractant protein-1) and the T cell chemoattractant CCL5, as well as CX3CR1, the receptor for CX3CL1 (fractalkine) expressed on monocyte-derived macrophages and required for their homeostasis (52, 53). Whereas the expression of *Ccl2* and *Ccl5* showed only marginal induction in both wildtype and *Nlrc5^-/-^* livers, *Cx3cr1* was strongly upregulated in both groups (**Figure 6A**). Next, we examined the gene expression of macrophage markers CD68 and F4/80 (ADGRE1) and T lymphocytes markers CD3ϵ and CD8α. As shown in **Figure 6B**, the fibrotic livers of both control and NLRC5-deficient mice showed increased expression of *Cd68* and *Adgre1*, and the latter was significantly higher in *Nlrc5^-/-^* livers. Whereas F4/80 is highly expressed in tissue-resident macrophages, CD68 is expressed in both tissue-resident and infiltrating macrophages (54, 55). The T cell marker transcript levels were not markedly altered by CCl_4_ treatment in both groups of mice. These findings suggest that NLRC5 deficiency increases the activation of liver-resident macrophages, which presumably contributes to elevated *Tnf* expression.

### NLRC5-Deficient Livers Display Elevated Levels of p65 Activation

**Figure 7:**
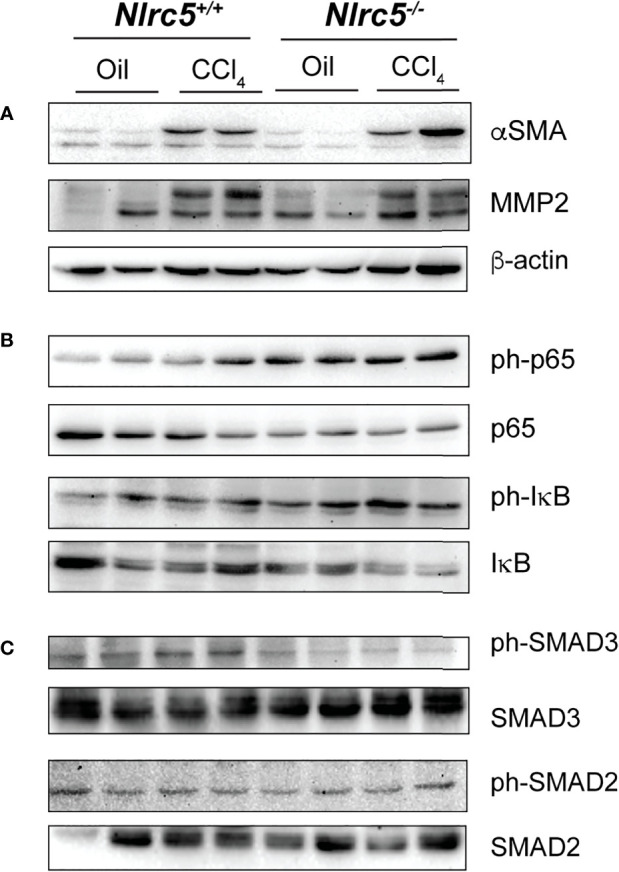
NLRC5-deficient livers display elevated levels of phospho-p65 and diminished levels of phospho-SMAD3. Liver tissue homogenates from control and NLRC5 -deficient livers following treatment with CCl4 or corn oil were evaluated for the expression of the indicated proteins associated with liver fibrosis **(A)**, NF-κB signaling **(B)** and TGFβ signaling **(C)**. At least four samples for each group from more than two experiments were tested, and representative data for two mice per group are shown.

Finally, we examined the protein expression of molecules associated with fibrosis and signaling events reported to be regulated by NLRC5 in whole liver homogenates. CCl_4_-treated wildtype and *Nlrc5^-/-^* mice livers showed increased levels of αSMA and MMP2 compared to vehicle-treated control groups (**Figure 7A**), reflecting the increased transcript levels of *Acta2* and *Mmp2* genes in the fibrotic livers (**Figures 3A**, **4B**). Notably, phosphorylation of the p65 subunit of NF-κB, which occurs downstream of diverse inflammatory signaling pathways including TNF (56), was found to be elevated in vehicle-treated *Nlrc5^-/-^* mice livers compared to wildtype control mice and this p65 phosphorylation was sustained following CCl_4_ treatment, with a concomitant decrease in total IκB (**Figure 7B**). This observation is consistent with the findings in the HSC cell line LX-2 following NLRC5 knockdown (37). However, phosphorylation of SMAD3, which occurs downstream of TGFβ signaling and reported to be reduced by NLRC5 knockdown in LX-2 cells (36), was reduced in *Nlrc5^-/-^* mice livers with or without CCl_4_ treatment, whereas phosphorylation of SMAD2 was comparable to control mice livers (**Figure 7C**). These results indicate that NLRC5 deficiency deregulates NF-κB activation and may also modulate the SMAD signaling pathway in the liver.

## Discussion

The growing healthcare burden of fibrotic diseases can be partly attributed to increased lifespan and the associated inflammaging as well as various lifestyle factors such as obesity and alcohol overuse. In addition to these factors, the limited progress in therapeutic control of the fibrogenic cascade has strengthened the efforts to understand the various molecular players with the goal of identifying potential pharmacological targets (5–7, 14, 57–60). Even though C57BL/6 mice are less susceptible than Balb/c mice to CCL_4_-induced liver fibrosis, various gene knockout mice in the C57BL/6 background have immensely contributed to the molecular understanding of liver fibrosis pathogenesis (61). Inflammatory cytokines such as TNFα and the fibrogenic cytokine TGFβ play key roles in the pathogenesis of liver fibrosis (41, 42, 62–64). IFNγ, which exerts antifibrogenic activity (65, 66), is a strong inducer of NLRC5 (67). The reports on NLRC5-mediated regulation of NF-κB and SMAD activation downstream of TNFα and TGFβ, respectively, in the human HSC cell line LX-2 raised the possibility that NLRC5 could be an important regulator of liver fibrosis and NLRC5-deficient mice would be useful to identify and characterize new drug targets to treat liver fibrosis. Our findings indicate that even though NLRC5 likely regulates these signaling events in the liver at steady state and after tissue injury, loss of these NLRC5-mediated regulatory mechanisms does not exacerbate liver fibrosis.

Our finding that NLRC5-deficient livers show increased phosphorylation of p65/RelA concurs with the previous reports on the regulatory functions of NLRC5 on NF-κB, although there are controversies about its universality (33). Initial studies showed that LPS-induced NF-κB activation was attenuated by NLRC5 overexpression whereas an inverse effect was observed by siRNA-mediated knockdown of NLRC5 in HEK293T cells expressing TLR4, in the murine macrophage cell line RAW264.7 and in mouse embryonic fibroblasts (MEF) (28, 29, 34). Mechanistically, NLRC5 mediated this inhibition by interacting with IκB kinases IKKαβ, thereby preventing them from being activated by NEMO downstream of LPS-induced TLR4 signaling (29). This inhibition was reported to be dynamically regulated by LPS-induced K63-linked polyubiquitination of NLRC5 and its deubiquitination by USP14 (29, 68). Subsequent studies using bone marrow-derived macrophages (BMDM), dendritic cells (BMDC) and peritoneal macrophages from four independently generated *Nlrc5^-/-^* mice showed that NLRC5 deficiency did not affect LPS-induced inflammatory cytokine production, although Tong et al., reported increased NF-κB activation and TNFα production in MEFs and BMDM following LPS stimulation (69–72). It has been suggested that differential ubiquitination of NLRC5 in different cell type may account for such differences (68). Nonetheless, elevated levels of phospho-p65 in NLRC5-deficient livers (**Figure 7B**) and increased expression of TNF following fibrosis induction (**Figures 5A, B**) confirm NLRC5-mediated regulation of NF-κB *in vivo*. This regulation may occur in hepatic macrophages, stellate cells and hepatocytes as all of them respond to TLR agonists (73). This possibility is supported by the elevated transcript levels of the tissue-resident macrophage marker F4/80 (*Adgre1*) (54) in the fibrotic livers of NLRC5-deficient mice (**Figure 6B**). NF-κB is also activated by TNFα (56) and both TLR and TNFα signaling pathways converge on the IKKαβγ complex regulated by NLRC5 (56, 68, 73). Thus, the elevated levels of phospho-p65 observed in NLRC5-deficient livers could result from both gut-derived TLR agonists and the resultant induction of TNFα in hepatic macrophages.

Intriguing differences were observed between NLRC5 knockout and wildtype mice livers in the induction of genes coding for the ECM modulating enzymes. Whereas *Mmp2* and *Timp2* genes are upregulated following CCl_4_ treatment in both wildtype and NLRC5-deficient livers, *Mmp3* and *Timp1* genes were not significantly induced in the absence of NLRC5 (**Figure 4B**). TIMP1 is an inhibitor of MMPs and thus promotes fibrogenesis but is not required to induce liver fibrosis (74). Hence, the reduced *Timp1* expression in NLRC5-deficient mouse livers is non-consequential on fibrosis development. However, *Timp1* is known to be induced by TNFα (75), and hence reduced Timp1 transcript levels in NLRC5-deficient mouse livers despite elevated levels of TNFα and NF-κB activation is intriguing.

Even though NLRC5 does not directly activate inflammasomes, it is reported to interact with NLRP3 and contribute to inflammasome activation and IL-1β production in the human monocyte cell line THP-1 (76). However, peritoneal macrophages from NLRC5 knockout mice did not show any change in IL-1β production compared to wildtype macrophages (69, 72). Besides, IL-1β does not figure predominantly in the pathogenesis of chronic liver diseases including liver fibrosis (77). Negligible changes in *Il1b* transcript levels (**Figure 6A**) and comparable level of liver fibrosis in NLRC5-deficient livers (**Figure 2**) suggest that NLRC5-dependent NLRP3 inflammasome activation plays little pathogenic role in liver fibrosis induced by chemically induced hepatocyte injury.

IFNγ is considered an anti-fibrogenic cytokine in the liver, but strain-dependent differences and pro-fibrogenic role in certain experimental models have been reported (65, 66, 78, 79). In the liver, IFNγ is produced by activated NK cells and T cells. Whereas IFNγ expression is significantly downmodulated following CCl_4_ treatment in wildtype mice livers, and opposite trend was observed in NLRC5-deficient mice. The reduced *Ifng* transcript levels in vehicle-treated *Nlrc5^-/-^* mice and its upregulation following fibrogenic stimuli suggest that NLRC5-dependent MHC-I expression may modulate the activation of immune cells under sterile inflammatory settings.

Li and colleagues have implicated NLRC5 in regulating signaling pathways activated by the key fibrogenic cytokine TGFβ, as NLRC5 knockdown in LX-2 cells enhanced TGFβ-induced phosphorylation of the activating SMADs SMAD3 and SMAD2, and increased expression of αSMA and collagen 1α1 genes (36). We did not find increased SMAD phosphorylation in the livers of CCl_4_-treated NLRC5-deficient mice compared to wildtype mice although *Tgfb* gene was induced to a similar extent in both groups. On the other hand, SMAD3 phosphorylation was diminished in NLRC5-deficient livers (**Figure 7C**). Even though the relatively high proportion of hepatocytes (60-80%) in the liver could mask any small difference in protein expression and their modification in a small proportion of HSCs, comparable levels of fibrosis induction in NLRC5-deficient and wildtype mice argues against the possibility of NLRC5-mediated modulation of TGFβ response impacting hepatic fibrogenesis.

Overall, our findings support the regulatory role of NLRC5 on NF-κB activation and TNF expression and suggest that this function may have a homeostatic role in restraining hepatic cellular activation by gut-derived TLR ligands. However, this NLRC5-mediated regulation is neither sufficient nor essential to overcome strong inflammatory and fibrogenic signaling such as the one induced by chronic chemical injury, as NLRC5-deficient and wildtype control mouse livers develop comparable levels of fibrosis. It is possible that adaptive repair mechanisms might have attenuated the increased inflammatory response in NLRC5-deficient mice, obscuring its effect after 5 weeks of CCl_4_ treatment. Therefore, it will be worthwhile to evaluate the effect of NLRC5 deficiency at early stages of acute injury. As TNF signaling plays a crucial pathogenic role in obesity-associated hepatic inflammation and hepatocarcinogenesis (10), the constitutively elevated p65 phosphorylation NLRC5-deficient livers also warrants further investigations into possible regulatory functions of NLRC5 on NF-κB activation and TNF production under milder but chronic inflammatory conditions such as the one associated with diet-induced fatty liver disease and HCC development.

## Data Availability Statement

The original contributions presented in the study are included in the article/**Supplementary Material**. Further inquiries can be directed to the corresponding author.

## Ethics Statement

The animal study was reviewed and approved by Université de Sherbrooke Animal Ethics Committee (Protocol # 2018-2083, 359-18C).

## Author Contributions

SI, TK, and SR conceived the idea. SI obtained funding. SI, AQ, and AS designed the experiments, analyzed data and wrote the manuscript. FR, MC, and AG repeated certain experiments. All authors contributed to the article and approved the submitted version.

## Funding

This work was supported by the Canadian Institutes of Health Research project grant PJT-153255 to SI. AG is a recipient of a postdoctoral fellowship from FRQS. CR-CHUS is an FRQS-funded research center.

## Conflict of Interest

The authors declare that the research was conducted in the absence of any commercial or financial relationships that could be construed as a potential conflict of interest.

## Publisher’s Note

All claims expressed in this article are solely those of the authors and do not necessarily represent those of their affiliated organizations, or those of the publisher, the editors and the reviewers. Any product that may be evaluated in this article, or claim that may be made by its manufacturer, is not guaranteed or endorsed by the publisher.

## Abbreviations

ALT, alanine transferase; CCl_4_, carbon tetrachloride; ECM, extracellular matrix; HSC, hepatic stellate cells; MMP, matrix metalloproteinase; SMA, alpha smooth muscle actin; TIMP, tissue inhibitor of MMP.
