# Supplementary material for: NLRC5 Deficiency Deregulates Hepatic Inflammatory Response but Does Not Aggravate Carbon Tetrachloride-Induced Liver Fibrosis
Source: Front Immunol. 2021 Oct 12;12:749646. doi: 10.3389/fimmu.2021.749646 (PMC8546206; doi:10.3389/fimmu.2021.749646)
Supplement: Supplementary file 1 [file DataSheet_1.pdf]

Table S1: List of RT-qPCR primers used in this study.

Gene name	Gene ID	Sense primer	Anti-sense primer	Amplicon Size (bp)
*36B4**	NM_007475.5	TCTGGAGGGTGTCCGCAA	CTTGACCTTTTCAGTAAGTGG	154
Acta2	NM_007392.3	AGTAATGGTTGGAATGG	GTGTCGGATGCTCTTCAGG	185
*Adgre1***	NM_001355722.1	CTTTGGCTATGGGCTTCCAGTC	GCAAGGAGGACAGAGTTTATCGTG	165
Ccl2	NM_011333.3	CAGGTCCCTGTCATGCTTCT	GTGGGGCATTAACTGCAT	91
Ccl5	NM_013653.3	TGCAGAGGACTCTGAGACAGC	GAGTGGTGTCCGAGCCATA	149
Cd3e	NM_007648.5	TGGAGCAAGAATAGGAAGGC	CATAGTCTGGGTTGGGAACAG	115
Cd68	NM_001291058.1	GTGTCTGATCTTGCTAGGACC	TGTGCTTTCTGTGGCTGTAG	118
Cd8a	NM_001081110.2	CATCACTCTCATCTGCTACCAC	TTTTCTCTGAAGGTCTGGGC	98
Col1a1	NM_007742.4	CTCCCAGAACATCACCTATCAC	ACTGTCTTGCCCCAAGTTCCG	192
Col3a1	NM_009930.2	AAGTCAAGGAGAAAGTGGTCG	CAGTCTCCCCATTCTTTCCAG	179
Cx3cr1	NM_009987.4	GTTATTTGGGCGACATTGTGG	ATGTCAGTGATGCTCTTGGG	142
Ifng	NM_008337.4	CCTAGCTCTGAGACAATGAACG	TTCCACATCTATGCCACTTGAG	150
Il1b	NM_008361.4	TCCTGTGTAATGAAAGACGGC	TCCTGTGTAATGAAAGACGGC	127
Il6	NM_031168.2	AGTCCGGAGAGGAGACTTCA	TTGCCATTGCACAACTCTTT	132
Mmp2	NM_008610.3	CAAGTTCCCCGGCGATGTC	TTCTGGTCAAGGTCACCTGTC	171
Mmp3	NM_010809.2	GATGAACGATGGACAGAGGATG	AAACGGGACAAGTCTGTGG	149
Pdgfb	NM_011057.4	CCTGCAAGTGTGAGACAGTAG	CTTTCGGTGCTTGCCTTTG	146
Timp1	NM_011593.2	TTGCATCTCTGGCATCTGG	TGGTCTCGTTGATTTCTGGG	156
Timp2	NM_011594.3	CAGGAAAGGCAGAAGGAGATG	GATCATGGGACAGCGAGTG	149
Tgfb1	NM_011577.2	ATACGCCTGAGTGGCTGTCT	CTGATCCCGTTGATTTCCA	148
Tnf	NM_013693.3	CGTCGTAGCAAACCACCAAG	GAGATAGCAAATCGGCTGACG	210

* *36B4* (*Rplp0*); ** *Adgre1* (F4/80)

Table S2: List of antibodies used in this study.

Name	Supplier	Cat no.	Clone no.
**Western blot**			
αSMA	Cell Signaling Technology	19245S	D4K9N (Rabbit mAb)
MMP2	Santa Cruz Biotechnology	SC-13595	8B4
Ph-IκBα	Santa Cruz Biotechnology	SC-8404	B-9
IκBα	Santa Cruz Biotechnology	SC-371	Rabbit polyclonal
Ph-p65 RelA (Ser536)	Cell Signaling Technology	3033L	93H1
p65	Santa Cruz Biotechnology	SC-372	Rabbit polyclonal
Ph-SMAD3 (Ser423/425)	Cell Signaling Technology	9520S	C25A9
SMAD3	Cell Signaling Technology	9513S	Rabbit polyclonal
Ph-SMAD2 (Ser465/467)	Cell Signaling Technology	3108L	138D4
SMAD2	Cell Signaling Technology	3103S	L16D3
Beta actin	Cell Signaling Technology	4970S	13E5
**Immunohistochemistry**			
αSMA	Cell Signaling Technology	19245S	D4K9N (Rabbit mAb)
